# Supplementary material for: Predicting suicide death among veterans after psychiatric hospitalization using transformer based models with social determinants and NLP
Source: Sci Rep. 2025 Nov 28;15:43623. doi: 10.1038/s41598-025-27435-6 (PMC12698767; doi:10.1038/s41598-025-27435-6)
Supplement: Supplementary file 2 — Supplementary Information 2. [file 41598_2025_27435_MOESM2_ESM.docx]

# Appendix 1: NLP-extracted SBDH

To extract SBDH from EHR notes, we deployed an NLP system to identify 9 SBDH predictors from EHR notes, namely, social isolation, transition of care, barriers to care, financial insecurity, housing instability, food insecurity, violence, legal problems, substance abuse, along with their presence (yes or no or n/a) and period (current or history or n/a) attributes.

We also identified two additional predictors from EHR notes, making the total of 11 NLP-extracted predictors. The two predictors are pain, which has been recognized as risk factor of suicide, and psychiatric symptoms (e.g., stress, anxiety, and depression), which has been frequently included also as a SBDH domain.

We used the NLP system to extract SBDH from the following 7 note types: emergency department notes, nursing assessments, social work case notes, hospital admission notes, mental health notes, pain management, and discharge summaries from the observation window. Based on the attribute values, we grouped each SBDH prediction into one of the following 4 categories:

1) ‘Current’: Prediction of an SBDH with presence ‘yes’ and period ‘current’.

2) ‘History’: Prediction of an SBDH with presence ‘yes’ and period ‘not current’.

3) ‘No’: Prediction of an SBDH with presence ‘no’, regardless of the predicted period.

4) ‘Unknown’: Prediction of SBDH with all other presence-period combinations.

If there are multiple categories for a single SBDH predictor in a single day, we used the following order: Current > History > No > Unknown. This yielded 44 unique SBDH risk factors with their associated record dates (11 SBDH predictors X 4 categories).

# Appendix 2: Pretraining and Finetuning Details

In this study, we built a large language model pretrained on the longitudinal EHRs of ~5.7 million patients who were seen in the VHA between 1/1/2016 to 06/01/2019, and who had at least 1 clinical visit during the period. Using 4 Nvidia Tesla P40 GPU of 22 GB graphics memory capacity, the model was pretrained for more than 140 hours with more than 280k steps and batch size of 48 and finetuned for more than 24 hours.

The risk of overfitting is a common problem in suicide prediction. L2 regularization (ridge regression) was applied to reduces the variance of the model. A 7 fold random cross-validation was applied to the training set to select the best hyperparameters, including learning rate selected with log uniform random from range [1e-6,1e-4], the L2 regularization weight selected with uniform random from range [0.1,0.5], and batch size selected with random choice of 4 or 8. The best hyperparameter was selected from 100 random trials with scalable hyperparameter tuning tool ray-tune. An asynchyperband scheduler with grace period of 5 and reduction factor of 2 is used. The hyperparameter with highest area under precision recall curve was selected.

# Appendix 3: Importance of Different Predictors

We performed permutation testing to identify different predictors and measure their influence on model performance for predicting suicide. Specifically, we calculated risk importance score *IS* for each predictor *r* using the equation below:

$$IS\left( r \right)=\frac{\sum_{c_{i} \in C_{r}} P\left( c_{i} \right)-P(c_{i} \backslash\{r\})}{|C_{r}|}$$

Where $C_{r}$ is the set of all cases with predictor *r* during their observation windows, $P\left( c_{i} \right)$ is our model’s predicted suicide probability score when $c_{i}$ is the input with all predictors, $c_{i} \backslash\{r\}$ is the same set of predictors with predictor *r* removed, and $|C_{r}|$ is the cardinality. A higher importance score indicates a higher contribution to the predictive model. To ease the complexity, we grouped ICD codes into broad categories by the first 3 characters. To better understand the importance of different predictors, we ranked them by their risk importance scores. There are 2,099 unique predictors among 226 suicide deaths after psychiatric discharges in the test data. Important predictors were identified only for the 226 suicide deaths (case group) because of the computation complexity that grows exponentially for the control group

We found that diseases, procedures, and medications were the most important predictors, followed by demographics, NLP-extracted SBDH, and ICD-based SBDH (Supplementary Table 3). Unsurprisingly we found that previous suicide attempts, cocaine related disorders and nicotine dependence were at the top of the risk factor list. We also found that cocaine dependence with intoxication (ICD-10 code: F14.220) ranked in the top 10 predictors while cocaine dependence in remission (F14.21) ranked further down (768th) in the list. Such diversity may indicate the importance of splitting a general predictor like cocaine related disorders (F14) into more specific categories.

SBDH from clinical notes played an important role in suicide prediction. Top 5 NLP-extracted SBDH ranked between 40-65 while the top 5 SBDH from ICD codes ranked between 46-539. The comparatively higher ranking for NLP-extracted SBDH could be attributed to the fact that they captured both current and past information, whereas ICD-based SBDH were only limited to current information. Besides, Truong et al. found that the uptake of ICD-based SBDH has been slow, and clinician-entered codes may poorly represent the actual social needs of patients.^1^ Among NLP-extracted SBDH, barriers to care was the most important SBDH predictor. This is consistent with the finding of Tondo et al. that areas with a higher density of doctors had lower suicide rates.^2^ Housing instability also plays an important role as many mentions are documented in clinical notes and can be extracted using NLP.^3^ Regardless of the source (structured data or notes), we found that housing instability as one of the top 5 SBDH predictors. This is not surprising as Aquin et al. already demonstrated that supplementing psychotherapy with "housing first" (providing permanent private unit housing) can reduce suicidal behavior.^4^

# Appendix 4: Tables and Figures

**Supplementary Table 1.** PTFCodes for selecting psychiatric admissions.

| 33- GEM PSYCHIATRIC BEDS |
| --- |
| 72 – ALCOHOL DEPENDENCE TRMT UNIT |
| 73 – DRUG DEPENDENCE TRMT UNIT |
| 74 – SUBSTANCE ABUSE TRMT UNIT |
| 79 – SIPU (SPEC INPT PTSD UNIT) |
| 91 – EVAL/BRF TRMT PTSD UNIT (EBTPU) |
| 92 – GEN INTERMEDIATE PSYCH |
| 93 – HIGH INTENSITY GEN INPT |
| 94 – PSYCHIATRIC OBSERVATION |
| 37 – DOMICILLARY CHV |
| 39 – GENERAL CWT/TR |
| 85 – DOMICILLIARY |
| 86 – DOMICILLARY SUBSTANCE ABUSE |
| 88 – DOMICILLIARY PTSD |
| 89 – STAR I, II, & III |
| 109 – PSYCH RESID REHAB PROG |
| 110 – PTSD RESID REHAB PROG |
| 111 – SUBSTANCE ABUSE RESID PROG |

**Supplementary Table 2.** Sociodemographic characteristics in this study.

| Characteristics | | Train  (n=138259) | Test  (n=59322) |
| --- | --- | --- | --- |
| Gender | Male | 127751 (92.4) | 54338 (91.6) |
|  | Female | 10508 (7.6) | 4984 (8.4) |
| Age | 18-29 | 6860 (5.0) | 3559 (6.0) |
|  | 30-39 | 31191 (22.6) | 12694 (21.4) |
|  | 40-49 | 21789 (15.8) | 9135 (15.4) |
|  | 50-59 | 30569 (22.1) | 14889 (25.1) |
|  | 60-69 | 29822 (21.6) | 11745 (19.8) |
|  | 70-79 | 15955 (11.5) | 6052 (10.2) |
|  | 80+ | 2073 (1.5) | 1248 (2.1) |
| Race | White | 95676 (69.2) | 40694 (68.6) |
|  | Black | 28481 (20.4) | 12396 (20.9) |
|  | Other | 12305 (8.8) | 5577 (9.4) |
|  | Asian | 1797 (1.3) | 655 (1.1) |
| Martial | divorced | 46842 (33.9) | 18508 (31.2) |
|  | married | 36391 (26.3) | 18330 (30.9) |
|  | never married | 39417 (28.5) | 16788 (28.3) |
|  | separated | 11821 (8.5) | 4211 (7.1) |
|  | other | 3788 (2.7) | 1485 (2.5) |
| Income Group* | 1 | 13618 (9.8) | 5635 (9.5) |
|  | 2 | 14586 (10.5) | 6347 (10.7) |
|  | 3 | 24959 (18.1) | 11330 (19.1) |
|  | 4 | 11669 (8.4) | 4923 (8.3) |
|  | 5 | 10742 (7.8) | 4271 (7.2) |
|  | 6 | 8834 (6.4) | 3618 (6.1) |
|  | 7 | 5696 (4.1) | 2669 (4.5) |
|  | missing | 48155 (34.8) | 20529 (34.6) |

Footnote

*: Income group number is calculated in the formula: (income_in_dollars-1.0)//8000+2. We treat “missing” as a categorical variable, like other groups.

**Supplementary Table 3.** Patient demographic distribution by gender and ethnicity.

| Ethnic Category | Female | Male | Total |
| --- | --- | --- | --- |
| Hispanic or Latino | 1936 | 16202 | 18138 |
| Not Hispanic or Latino | 13082 | 159883 | 172965 |
| Unknown | 474 | 6004 | 6478 |
| Total | 15492 | 182089 | 197581 |

**Supplementary Table 4.** Area under the receiver operating characteristic curve (AUROC) for suicide death prediction within 180 days after psychiatric discharges among different Race and Age group.

|  |  | **Model with SBDH** | **Model without SBDH** |
| --- | --- | --- | --- |
| **Race** | **White** | **64.1** | **62.4** |
|  | **Black** | **63.9** | **61.6** |
|  | **Other** | **63.7** | **61.3** |
|  | **Asian** | **62.9** | **60.1** |
| **Age** | **18-29** | **62.9** | **60.5** |
|  | **30-39** | **63.7** | **61.8** |
|  | **40-49** | **64.5** | **62.7** |
|  | **50-59** | **64.3** | **62.5** |
|  | **60-69** | **64.4** | **62.7** |
|  | **70-79** | **63.1** | **60.9** |
|  | **80+** | **63.4** | **60.4** |

**Supplementary Table 5.** Top 5 important predictors which lead to suicide death. These predictors are ranked out of 557 unique predictors and 226 suicide death cases.

| Rank | Code | Description | Cases |  |
| --- | --- | --- | --- | --- |
| Diseases and Procedures | | | | |
| 1 | T14 | Suicide attempt | 14 |  |
| 2 | J45 | Asthma | 2 |  |
| 6 | F14 | Cocaine related disorders | 62 |  |
| 17 | F17 | Nicotine dependence | 24 |  |
| 18 | K92 | Other diseases of digestive system | 3 |  |
| Medications | | | | |
| 3 | 4017919 | AMOXAPINE | 3 |  |
| 4 | 4037714 | MACIMORELIN | 2 |  |
| 5 | 4021046 | IVERMECTIN | 34 |  |
| 7 | 4019440 | NICOTINIC ACID | 11 |  |
| 8 | 4019714 | DICLOFENAC | 13 |  |
| Demographics | | | | |
| 13 | RAC-B | Race Black | 26 |  |
| 52 | AGE-3 | 29 < Age < 40 | 63 |  |
| 60 | RAC-U | Race Unknown | 15 |  |
| 73 | INC-1 | Income = 0 | 24 |  |
| 98 | INC-5 | $24,001 < Annual income < $32,001 | 11 |  |
| SBDH from ICD | | | | |
| 46 | Z72 | Problems related to lifestyle | 25 |  |
| 113 | Z59 | Problems related to housing and economic circumstances | 116 |  |
| 155 | Z65 | Problems related to other psychosocial circumstances | 194 |  |
| 281 | Z63 | Problems related to support, including family circumstances | 17 |  |
| 539 | Z56 | Problems related to employment and unemployment | 53 |  |
| SBDH from note | | | | |
| 40 | BARRIERSTOCARE1 | Currently difficulty to access care | 136 |  |
| 54 | TRANSITIONSOFCARE1 | Currently change in medication, transfer etc. | 219 |  |
| 61 | HOUSINGINSTABILITY1 | Currently experience in eviction, homeless, homelessness | 214 |  |
| 62 | PSYCHIATRICSYMPTOMS1 | Currently have psychiatric symptoms | 117 |  |
| 65 | PAIN1 | Currently suffer from pain or discomfort | 187 |  |

**Supplementary Figure 1.** Calibration analysis of logistic regression, ensemble, and TransformEHR with SBDH on the testing dataset.


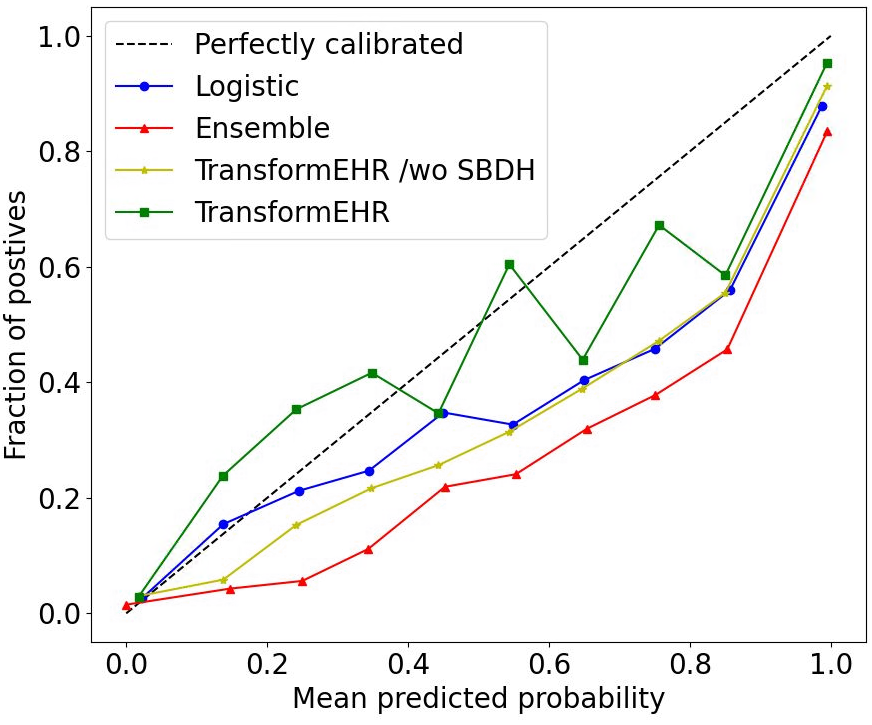


**Supplementary Figure 2.** Area under the receiver operating characteristic curve (AUROC) among patients from 129 hospitals. Hospitals are grouped by the number of patients in the training data. There are 1530 patients in a hospital on average. The AUROC is consistent among different hospitals, even for those hospitals which had no patients in the training data.


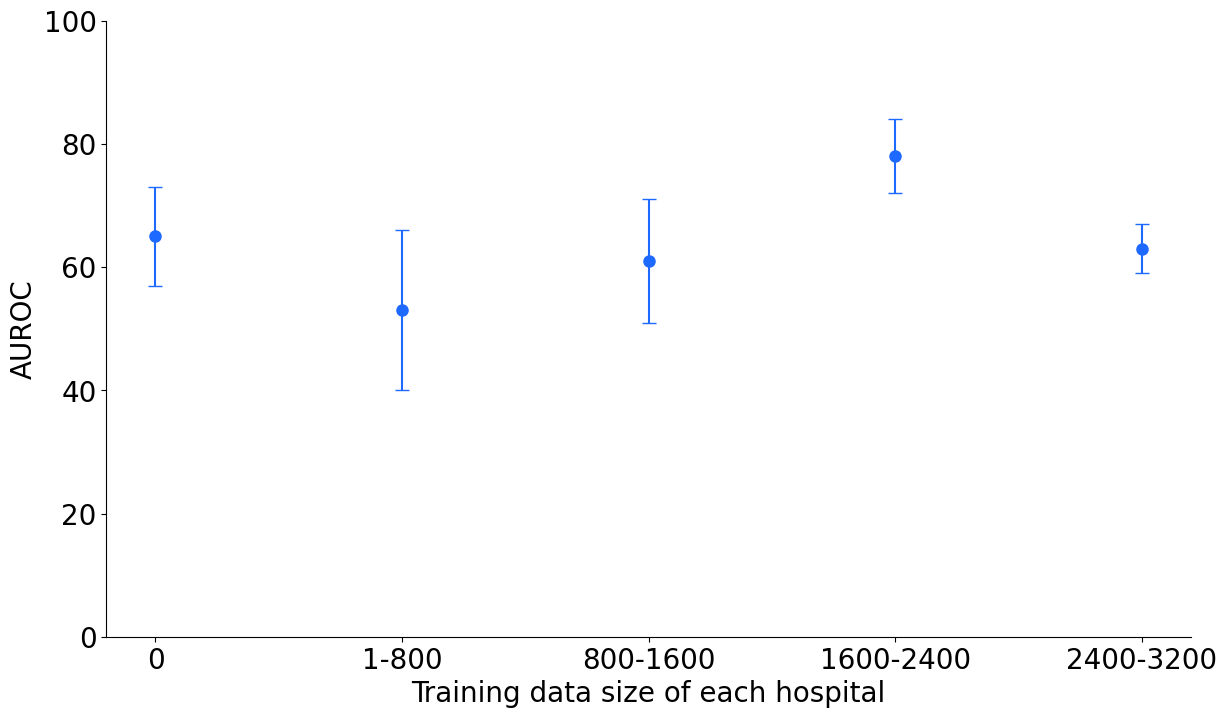


**Supplementary Figure 3.** Area under the receiver operating characteristic curve (AUROC) for suicide death prediction within 180 days after psychiatric discharges among Males and Females.


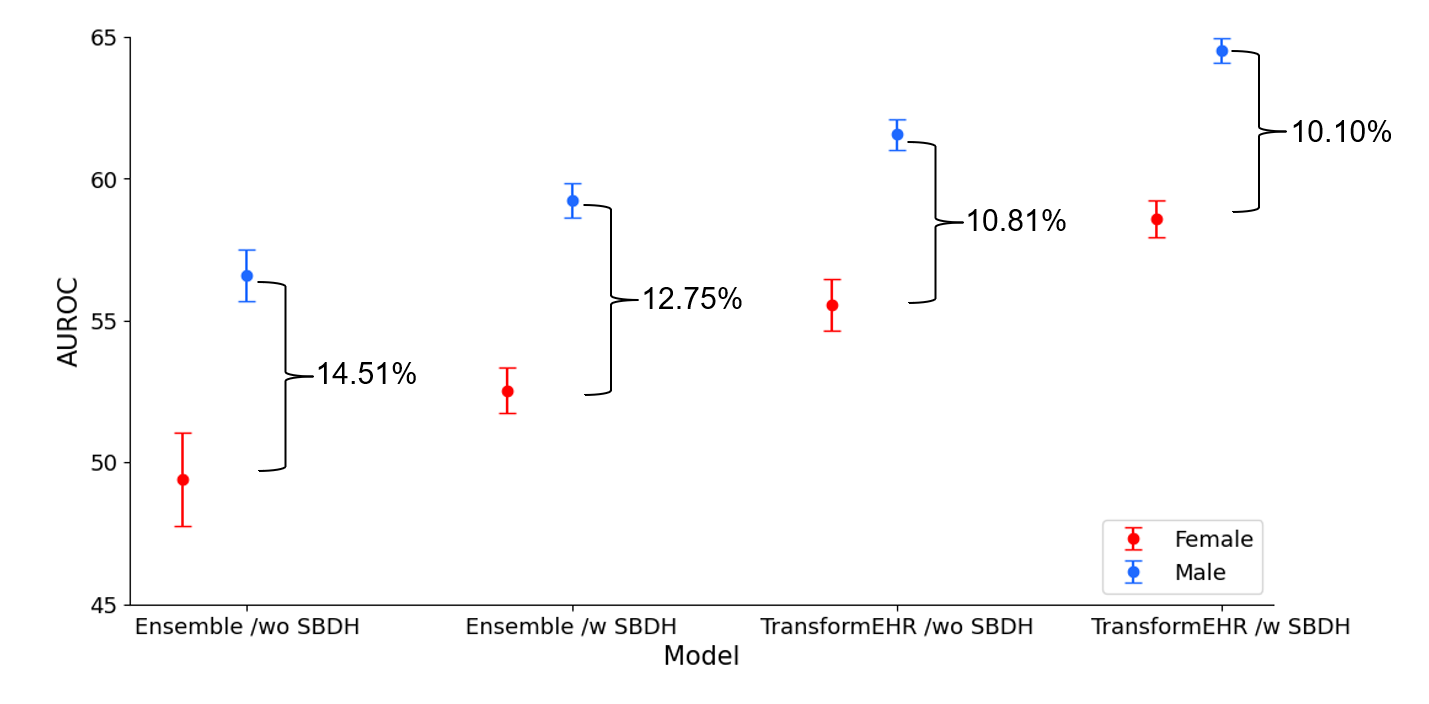


# References

1. Truong HP, Luke AA, Hammond G, Wadhera RK, Reidhead M, Maddox KEJ. Utilization of Social Determinants of Health ICD-10 Z-Codes Among Hospitalized Patients in the United States, 2016–2017. *Med Care*. 2020;58:1037-1043.

2. Tondo L, Albert M, Baldessarini RJ. Suicide rates in relation to health care access in the United States: an ecological study. *J Clin Psychiatry*. 2006;67 4:517-523.

3. Chapman AB, Scharfstein DO, Montgomery AE, et al. Using natural language processing to study homelessness longitudinally with electronic health record data subject to irregular observations. *AMIA Annu Symp Proc AMIA Symp*. 2023;2023:894-903.

4. Aquin JP, Roos LE, Distasio J. Effect of Housing First on Suicidal Behaviour: A Randomised Controlled Trial of Homeless Adults with Mental Disorders. *Can J Psychiatry*. 2017;62(7):473-481. doi:10.1177/0706743717694836
